# Loss of inter-cellular cooperation by complete epithelial-mesenchymal transition supports favorable outcomes in basal breast cancer patients

**DOI:** 10.18632/oncotarget.25034

**Published:** 2018-04-13

**Authors:** Anne Grosse-Wilde, Rolf E Kuestner, Stephanie M Skelton, Ellie MacIntosh, Aymeric Fouquier d’Hérouël, Gökhan Ertaylan, Antonio del Sol, Alexander Skupin, Sui Huang

**Affiliations:** ^1^ Institute for Systems Biology, Seattle, WA, USA; ^2^ Luxembourg Centre for Systems Biomedicine, Esch-sur-Alzette, Luxembourg; ^3^ Environmental Risk and Health Unit, Flemish Institute for Technological Research (VITO), Mol, Belgium

**Keywords:** complete EMT, metastasis, cooperation, breast cancer, patient survival

## Abstract

According to the sequential metastasis model, aggressive mesenchymal (M) metastasis-initiating cells (MICs) are generated by an epithelial-mesenchymal transition (EMT) which eventually is reversed by a mesenchymal-epithelial transition (MET) and outgrowth of life-threatening epithelial (E) macrometastases. Paradoxically, in breast cancer M signatures are linked with more favorable outcomes than E signatures, and M cells are often dispensable for metastasis in mouse models. Here we present evidence at the cellular and patient level for the cooperation metastasis model, according to which E cells are MICs, while M cells merely support E cell persistence through cooperation. We tracked the fates of co-cultured E and M clones and of fluorescent CDH1-promoter-driven cell lines reporting the E state derived from basal breast cancer HMLER cells. Cells were placed in suspension state and allowed to reattach and select an EMT cell fate. Flow cytometry, single cell and bulk gene expression analyses revealed that only pre-existing E cells generated E cells, mixed E/M populations, or stem-like hybrid E/M cells after suspension and that complete EMT manifest in M clones and CDH1-negative reporter cells resulted in loss of cell plasticity, suggesting full transdifferentiation. Mechanistically, E-M coculture experiments supported the persistence of pre-existing E cells where M cells inhibited EMT of E cells in a mutual cooperation via direct cell-cell contact. Consistently, M signatures were associated with more favorable patient outcomes compared to E signatures in breast cancer, specifically in basal breast cancer patients. These findings suggest a potential benefit of complete EMT for basal breast cancer patients.

## INTRODUCTION

While most primary breast tumors are successfully eliminated by surgery, metastasis remains incurable and accounts for the vast majority of patient deaths [1]. Metastatic tumors are thought to originate from a small population of stem-like cells within the primary tumor, termed cancer stem cells (CSCs), which due to their capacity of self-renewal and plasticity [2] can initiate the metastatic cascade requiring invasion, migration, intravasation, survival of detachment and anoikis resistance in the circulation, extravasation and formation of epithelial macrometastases. Accordingly, CSC enrichment in primary tumors can predict poor patient outcomes [3]. The notion of the CSC-concept was soon combined with the older idea of EMT, during which epithelial (E) carcinoma cells with cobble-stone like morphology convert to a fibroblast-like mesenchymal (M) type of cell that can complete the initial steps of the metastatic cascade. Indeed, in the context of luminal epithelial cell lines of mammary gland tissue or tumors, such as HMLE, HMLER, and MCF7, the M cell-type has more CSC-like properties than the epithelial bulk population [4, 5]. However, in the context of basal, mesenchymal cell lines, more CSC-like properties were detected in the adhesion–dependent E population [6–9]. The concept of stemness of an intermediate E/M state can explain context-dependency and has been supported experimentally at the cell population level indicating a mixture and cooperation of E and M cells and is manifested at single cell level in the existence of a hybrid E/M cell type that co-expresses E and M gene signatures *in vitro* and *in vivo* [10–15], and has been predicted by theoretical models [16]. Consistently, co-expression of E and M-specific gene signatures in patient tumors, either due to mixture or presence of the hybrid cells, predicts poor survival in diverse breast cancer subtypes [12]. However, to date the stem-like intermediate E/M state remains untargetable due to the absence of specific markers, in comparison to the better defined differentiated E or differentiated M states, and the cellular origin of hybrid E/M cells remains unclear.

Previously, two competing metastasis models have been proposed, where metastases are either caused by (1) individual M cells establishing new metastatic tumors (as CSCs or MICs) according to the popular *sequential metastasis model* or (2) by E cells acting as MICs with cooperating M cells as supporting cells, as proposed by the *cooperation metastasis model*.

The *sequential metastasis model* (1) assumes that the metastatic process is initiated by an EMT [17], generating individual aggressive M cells [18]. Since life-threatening proliferating macrometastases typically have epithelial morphology and are carcinoma, often exhibiting features of normal differentiated breast epithelium, it has been postulated that for colonization and expansion at the new site the individual M cell must reverse to the epithelial state in a process referred to as mesenchymal-to-epithelial-transition (MET) [17, 19, 20]. This process implies plasticity of M cells. However, experimental validation of complete MET of individual cells *in vivo* is still lacking [21, 22]. In support of MET, or reversibility of EMT, we recently demonstrated *in vitro* that clonal M cells from the tumorigenic breast cell line HMLER cultured as stem cell enriched mammospheres (MS) could undergo partial MET and generated individual hybrid E/M cells [12], but their stability remained unclear. However, several experimental observations suggest that complete EMT is irreversible because sustained and complete EMT induction ablates cellular phenotypic plasticity *in vitro* [9, 23–27]. Accordingly, in mice continuous induction of EMT decreases incidence of epithelial metastasis [26, 28]. Further, *in vitro* findings show that single cell-derived M clones from HMLER cells are not plastic [12, 29]. Finally, *in vivo* cell tracking in mice revealed that EMT and thus M cells did not form lung metastases in breast and pancreatic cancer [30, 31], further questioning if M cells are MICs.

Consistent with the observed absence of M cell plasticity, the alternative *cooperation metastasis model* (2), originally termed cooperativity theory [32], proposes that M cells mainly support E cells by cell-cell cooperation, and that epithelial metastases are directly derived from pre-existing E cells, implying that MICs are epithelial cells. Hence, metastasis would not require MET plasticity of individual M cells. Direct support for the cooperation metastasis model comes from reports that in mice coinjection of E and M cells increases distant metastasis formation derived from pre-existing E cells [6, 33, 34]. Thus, increased stemness and mammosphere formation of cooperating HMLER E and M cells, and of the mixed E/M state at the population level are consistent with the cooperation metastasis model [12]. The intriguing consequence of M cells being merely supporting cells for E MICs has not been examined in detail yet but would suggest that successful therapeutic induction of complete EMT beyond the intermediate E/M state might transdifferentiate epithelial cancers into a non-cancer M state, and possibly irreversibly eliminate E MICs. However, the cellular mechanism for how cooperation between E and M cells prevents detachment-induced anoikis and EMT plasticity of E cells remains unclear.

To directly contrast the two metastasis models with either M or E cells being MICs, we combined *in vitro* and *in silico* strategies. In the *in vitro* studies using clonal E and M monocultures and E/M cocultures we studied whether upon detachment it is the M cells that underwent MET or the E cells that persisted and resisted detachment-induced anoikis, thereby initiating premetastatic E populations. To this end, we used the breast-derived heterogeneous basal HMLER cell line [29, 35], that contains both E (CD24+/CD44–) and M (CD24–/CD44+) cell populations. Using publicly available breast cancer patient data, we compared the association of E versus M signatures with poor survival and metastases.

Together both our *in vitro* and patient data are in line with the cooperation metastasis model: we show *in vitro* that pre-existing E cells were required to generate E subpopulations and were supported by cooperation with transdifferentiated M cells. Consistently, in breast cancer patients, the expression of E signatures alone or together with M signatures predicted worse or equal outcomes than pure M signatures, specifically for basal breast cancer patients, suggesting that E cells are the MICs.

## RESULTS

### The suspension-induced intermediate E/M gene expression state is unstable in M cells upon readhesion, but stable in E cells

**Figure 1:**
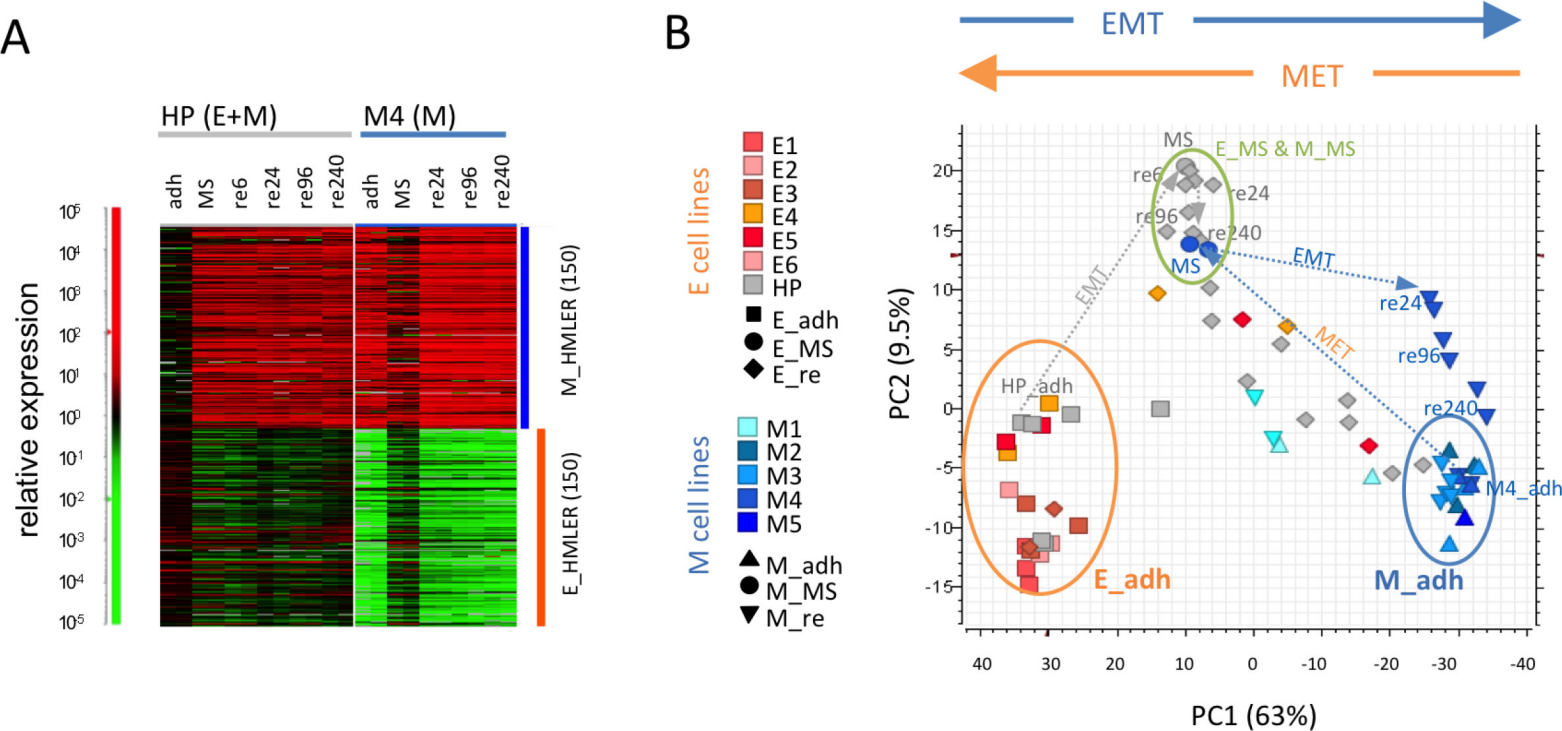
Stability of the intermediate E/M state in epithelial but not in mesenchymal cell lines (**A**) Heatmap of relative gene expression as measured in biological replicate arrays from HP and M4 cells that were grown in adhesion (adh), suspension as mammospheres (MS) and replated for the indicated times from 6 to 240 hours (re6, re24, re96, re240). Expression of indicated E_HMLER and M_HMLER (150) signatures are shown. (**B**) Principal component analysis (PCA) of gene expression arrays from M4 and HP cell lines in (A) and additional HP cell replicates, HMLER-derived E and M single cell clones cultured in adhesion and after replating using E_HMLER (150) and M_HMLER (150) signatures. Arrows in gray (HP) and blue (M4) indicate state changes of populations between adhesion, mammospheres and replating. All experiments shown were done in biological replicates (at least duplicates).

To form macrometastases cancer cells need to detach from the primary tumor, survive in suspension, and eventually readhere to a secondary site and express epithelial genes. To test the potential of M cells to undergo MET during the metastatic process, we used (bulk) mRNA expression profiling to assess the stability of the intermediate E/M state generated by M cells during suspension mammosphere culture, using the single cell-derived HMLER M clone M4, which only contained CD24–/CD44+ (M) cells but no CD24+/CD44– (E) cells [12]. For comparison, and to test the alternative cooperation metastasis model with E cells as MICs, we tested the parental HMLER cell line (HP) containing mostly CD24+/CD44– E cells under the same conditions. As recently demonstrated, both HP and M4 cells converged to the intermediate E/M state as assessed by gene expression analysis using 150 previously defined epithelial (E_HMLER) and mesenchymal (M_HMLER) specific gene sets [12] (Figure 1A). Since life-threatening metastases are epithelial and adherent we reasoned that replating to adhesion culture (Supplementary Figure 1A) should maintain at least some E signature expression and an intermediate E/M state. Surprisingly, already 24 hours following reattachment (and thereafter) the intermediate E/M mammosphere cultures derived from M4 cells had completely lost the E signature and regained pure M signature gene expression. This was suggestive of complete EMT of M4 cells upon readhesion back into an M state resembling that of the adherent source M cells from the M4 clone (Figure 1A). By contrast, replated HP-derived mammosphere cultures did not change gene expression pattern relative to that of the suspension cultures, and maintained their intermediate E/M state when analyzed at 6 hours, 24 hours and even ten days past replating.

To test the validity of these observations for other cell lines, we compared the gene expression profiles of adherent versus replated cultures of multiple HMLER M and E clones, as well as HP cells of different freezing passages. Gene expression profiles of these cells are visualized in a principal component analysis (PCA) that uses E and M signature gene expression values (Figure 1B). HMLER_E and HMLER_M signatures were originally defined by genes that discriminate most strongly between adherent HMLER E and M clones. The PCA placed adherent E and M clones to opposite ends of principal component 1 (PC1), while suspension cultures of either origin were located in the intermediate state between E and M states. Interestingly, in the PCA the M4 clones displayed a rapid, replating-induced reversion back to the original M state along PC1, suggesting complete EMT and instability of the M-derived E/M state in suspension.

All additional M clones (M1, M2, M3, M5) exhibited the same trend and stably maintained their original M state with no increased E signature expression after replating (Figure 1B, Supplementary Figure 1B). As expected, suspension culture of E clones (E1 to E6) resulted in nearly no visible mammospheres compared to the M clones (Supplementary Figure 1A), consistent with anoikis of E cells [36] or quiescence upon detachment. Intriguingly, replating of each of the 6 tested E clone-derived cultures as well as each of the 11 mostly epithelial HP mammosphere cultures resulted in a viable readhesion culture that could be analyzed by gene expression analysis (Supplementary Figure 1A). In contrast to M clones, all biological replicates of 2 out of 3 tested E clones and all tested (epithelial) HP cell line replicates reached a stable intermediate E/M state due to partial loss of E signatures and gain of M signatures which is consistent with partial EMT after replating.

Taken together, these data suggested that HMLER M clones reside in a rather stable M state and the partial MET of M clones in suspension was only transient. By contrast, HMLER E cell lines consistently generated stable intermediate E/M populations upon suspension and replating. Thus, EMT was inevitable upon placing in suspension culture and replating of HMLER cells, which resulted in an increase of heterogeneity in E cell lines but loss of heterogeneity in M cell lines.

### Mesenchymal HMLER cell lines do not exhibit E/M heterogeneity when examined by single cell analysis

**Figure 2:**
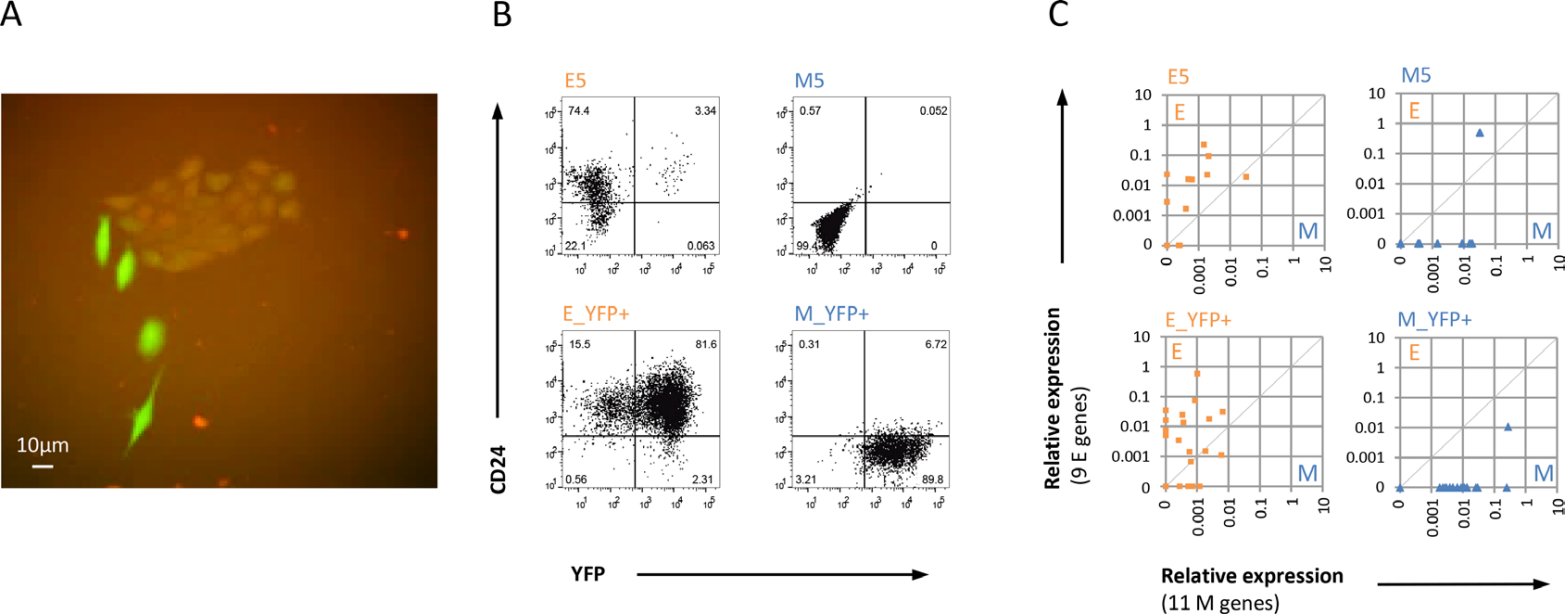
Abundance of hybrid E/M cells in E versus M HMLER cell lines (**A**) Overlay fluorescent microscopy shows heterogeneous HP cells transduced with dual reporter construct (CDH1-reporter) where red indicates the E state by active CDH1 promoter visualized by mCherry and green indicates live and transduced cells by active SV40 promoter shown by YFP (40× magnification). (**B**) FACS profiles of αCD24-stained non-transduced and CDH1 reporter transduced stable E and M cell lines. (**C**) Single cell qPCR analysis shows E/M state space representation of E5, M4, E_YFP+, and M_YFP+ cell populations using aggregate expression of 9 E genes and 11 M genes (see Methods). Note that E cell lines contain mostly hybrid E/M cells and M cell lines show complete EMT in most cells. Data are representative for both independent experiments using different reporter cell lines.

The population level gene expression data were consistent with our previous observations in single cell analyses of HMLER M clones that showed stable RNA expression of M signatures and absence of any E genes, while in HMLER E clones the majority cells individually co-expressed both E and M signatures indicating a hybrid E/M state [12]. To ensure that this finding was not due to potential clonal artifacts, we generated a lentiviral dual fluorescent reporter vector, designated CDH1 reporter, using the promoter for the gene *CDH1,* which encodes the epithelial adhesion protein E-Cadherin to drive expression of the red fluorescent mCherry (mCh) that reports the E state. The yellow fluorescence protein YFP expressed constitutively served as a positive control for presence of the reporter vector (Supplementary Figure 2A, Methods). As expected, CDH1 reporter-transduced mixed HP populations contained typical E colonies that co-expressed mCh and YFP, while cells classified as M cells based on morphology only expressed YFP (Figure 2A). Using FACS we then generated non-clonal trackable YFP-positive E and M cell lines by sorting for mCh+/YFP+ cells and mCh–/YFP+ cells (Supplementary Figure 2B) corresponding to CD24+ epithelial E_YFP+ and CD24− mesenchymal M_YFP+ cell lines, respectively. As expected, all E_YFP+ and M_YFP+ cell lines resembled HMLER E and M clones, respectively, in CD24-expression (Figure 2B) and morphology (Supplementary Figure 2C).

Next, we sorted individual YFP+ cells from the E_YFP+ and M_YFP+ cell lines passaged as adhesion cultures for single cell qPCR expression analysis of 9 E-specific and 11 M-specific marker genes. Results were plotted in an E/M state space (Figure 2C) combining the cell state specific markers (Methods). qPCR analysis of E_YFP+ cells confirmed the previously observed E/M heterogeneity of E5 and non-clonal HP cell lines [12] since 11 of 19 evaluable E_YFP+ cells exhibited a hybrid E/M signature (58%), with four cells (21%) exclusively expressing M genes and four cells exclusively expressing E genes (Figure 2C). By contrast, only one (5%) of the 20 evaluable single M_YFP+ cells showed a hybrid E/M signature, none expressed E genes, while all others (95%) exclusively expressed M genes, and thus resembled the M5 (Figure 2C) and M4 clones [12]. Thus, single cell analysis of nonclonal HMLER E and M reporter cell lines showed similarity with the respective clonal cell lines by sharing morphology, presence or absence of E/M heterogeneity, CD24 expression, and hybrid E/M status.

### Coculture with M cells allows for persistence of E cells during suspension culture and replating

**Figure 3:**
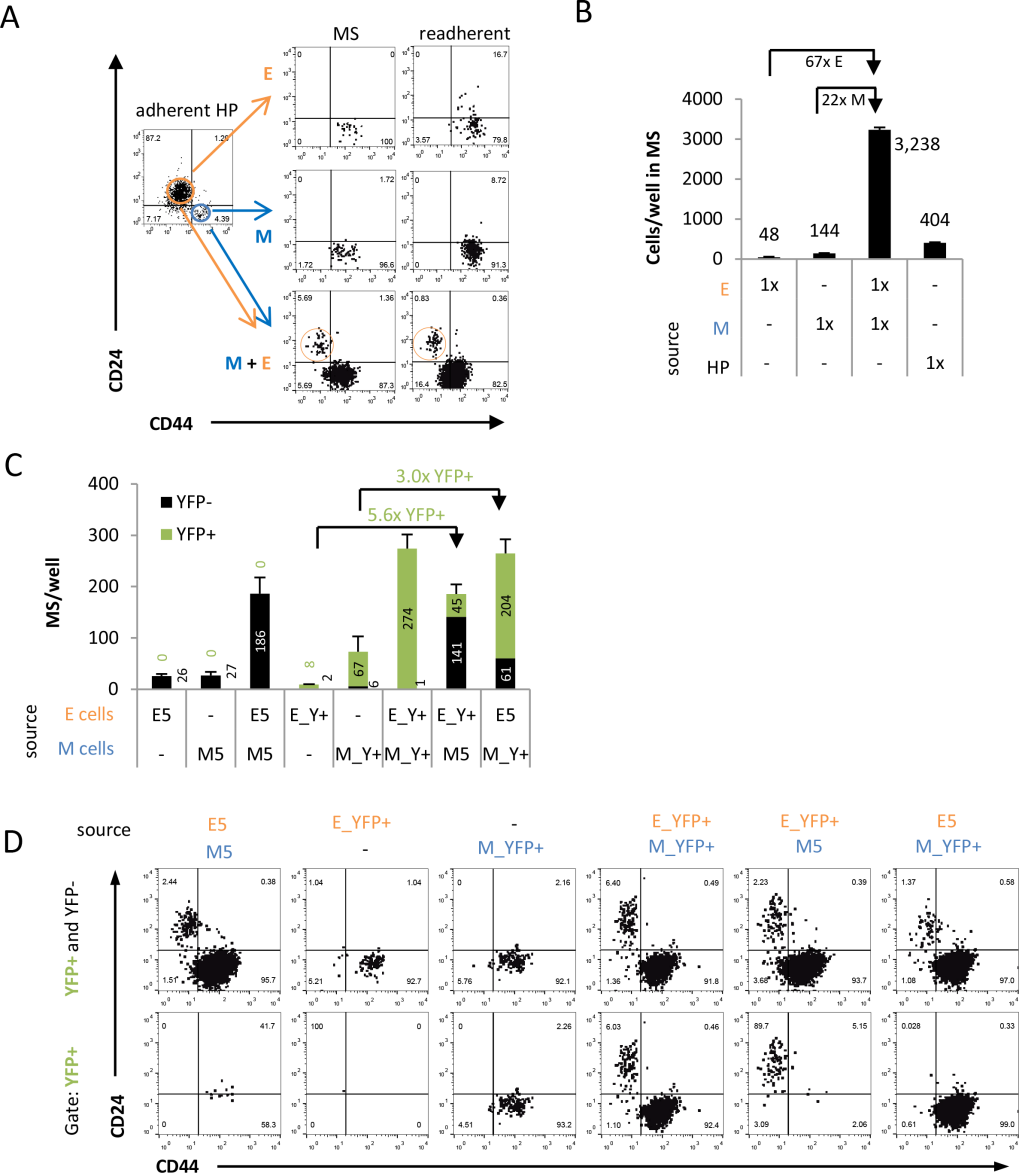
Coculture with M cells facilitates E cell persistence and inhibition of EMT in suspension (**A**) After suspension mammosphere culture condition (MS), E cells are only found in mammospheres derived from E/M cell cocultures but not from E or M monocultures. Quantitative flow cytometry profiles of sorted E (CD24+/CD44−), M (CD24−/CD44+) subpopulations and unselected HP cells cultured alone or together (1,000 cells per cell-type) were grown as mammospheres for two weeks, and subsequently replated to adhesion for another week. (**B**) Total surviving cell numbers from dissociated mammosphere cultures shown in (A). (**C**) Total number of YFP- and YFP+ mammospheres per well after two weeks suspension of E/M cocultures and E or M monocultures as assessed by flow cytometry. (**D**) Flow cytometry analysis of dissociated and αCD24/αCD44-stained mammosphere cultures of E/M cell cocultures from (C) either gated for live cells (YFP+ and YFP−) or live YFP+ cells (YFP+) with each column representing the same sample. Note that no YFP+/CD24+/CD44− cells were found in E/M cocultures with M_YFP+ cells, and no YFP+/CD24−/CD44+ cells were detected in E/M cocultures with E-YFP+ cells. All data are representative for experiments performed in biological duplicates.

When subjected to suspension culture, HMLER E (CD24+/CD44–) populations either undergo anoikis or EMT and become CD24–/CD44+ (M) cells, while HMLER M (CD24–/CD44+) cells are not plastic and do not undergo MET [12]. Thus, it remains unclear how E cells can form macrometastases at a distant site after a prolonged detachment phase. To investigate whether cooperation between cells could be responsible for survival of E cells after suspension and replating, we examined cocultures of E and M HMLER cells. Using flow cytometry, E and M cells from a heterogeneous HMLER cell line were sorted and cocultured in a 1:1 ratio under suspension mammosphere conditions for two weeks. After dissociation of mammosphere cultures, staining, and flow cytometry, intriguingly we detected a small but consistently distinct CD24+/CD44– (E) subpopulation in these cocultures (Figure 3A). By contrast, no E cells but only pure M cell populations were detected when E or M cells were cultured by themselves (Figure 3A). Even increasing the seeded cell number from 2,000 to 16,000 cells (Supplementary Figure 3A) did not induce an E cell subpopulation in the M clone-derived mammospheres, again suggesting the absence of spontaneous MET in M monocultures. Consistent with our previous findings of E and M cooperation [12], the 1:1 coculture resulted in increased total cell numbers compared to monoculture of E cells (more than 60-fold), M cells (more than 20-fold), and more than 16-fold compared to HP cultures (Figure 3B). Replating suspension cultures to adhesion culture, staining, and flow cytometry analysis confirmed that the E population found in cocultures was stably maintained through the two weeks of each suspension culture and the subsequent replating, while it remained absent in replated mammospheres of E or M monocultures (Figure 3A, Supplementary Figure 3A). Intriguingly, in the heterogeneous HP cells that were mostly E cells (72%) but even under adhesion culture contained a pre-existing M subpopulation of 18%, we consistently detected an E subpopulation after suspension and replating (Supplementary Figure 3A) suggesting that cooperation could occur also in the mixed HP cell population.

The presence of E cells in suspension cocultures, high E cell plasticity and absence of MET plasticity of M cells suggested that cooperation of E and M cells can facilitate survival, persistence, as well as readhesion of pre-existing E cells by cooperation with M cells indicating E cells being MICs. On the other hand, the findings could not exclude the possibility that coculture with E cells generates a suitable microenvironment for MET of M cells with M cells being MICs.

### Coculture of E and M cells results in persistence of E cells by suppression of epithelial plasticity

To identify whether E or M cells gave rise to the CD24+/CD44– (E) subpopulation under coculture conditions in suspension, we utilized the genetically YFP-labelled E_YFP+ and M_YFP+ cell lines. Consistent with single cell analyses (Figure 2) and with observations in HMLER E clones and freshly sorted CD24+/CD44– (E) cells from HP cells (Figure 3A), E_YFP+ cells were highly plastic, as they converted to the CD24–/CD44+ (M) state after mammosphere suspension culture. Of note, during suspension-induced EMT E_YFP+ cells lost YFP expression. By contrast, within the same period of two weeks M_YFP+ cells retained YFP, and stably remained in their CD24−/CD44+ (M) state (Figure 3D) consistent with absence of plasticity in M clones. E and M cocultures were generated by sorting 1:1 ratios of E_YFP+ cells and M_YFP+ cells or in either combination with the non-fluorescent E5 and M5 clones under suspension mammosphere conditions (Figure 3C, 3D, Supplementary Figure 3B, 3C). According to our observations of cooperation (Figure 3A), all E and M cell coculture combinations consistently contained a small distinct E cell subpopulation and both E and M cells expressed YFP in dissociated suspension cultures (Figure 3D). Moreover, all combinations of E and M cell cocultures showed a synergistic increase of number of mammosphere formed (Figure 3C) and of cell numbers in dissociated mammosphere (Supplementary Figure 3B) by at least 3-fold or 5-fold, respectively, relative to projected additive effect using numbers from E or M cell monocultures.

Tracking the origin of E cells in cocultures of E_YFP+ and unlabeled M5 cells exhibited an increased number of YFP+ mammospheres (5-fold) and YFP+/CD24+/CD44− (E) cells (30-fold) compared to E_YFP+ suspension culture alone (Figure 3C, Supplementary Figure 3B). In these cultures, 83% of all E cells were found to express YFP (Figure 3D, Supplementary Figure 3C). Conversely, in mammosphere cocultures of M_YFP+ with E5 cells no E cells were YFP+ (Figure 3D, Supplementary Figure 3C). This suggested that during suspension mammosphere culture the E subpopulation was derived from pre-existing, persisting E cells and not by MET from M cells, consistent with the hypothesis of an irreversibly transdifferentiated state of HMLER M cells.

When tracking the origin of M cells in suspension in cocultures of M_YFP+ cells with E5 cells, we observed an increase of YFP+ mammospheres (3-fold) and of YFP+/CD24−/CD44+ cell (18-fold) numbers compared to M_YFP+ cells cultured alone (Figure 3C, Supplementary Figure 3B). Nearly all CD24−/CD44+ (M) cells were YFP+ in cocultures of E5 and M_YFP+ cells (97%). But surprisingly, no M cells were found to be YFP+ in cocultures of E_YFP+ and M5 cells (Figure 3C, Supplementary Figure 3C). Hence, the highly plastic E cells did not convert to the M state by EMT when cocultured in suspension with M cells. Together these data suggested that the mechanism of cooperation between E and M cells involves *inhibition* of E cell plasticity and is accompanied by proliferation of M cells.

Besides the independent validation of absence of plasticity of HMLER M cells, cell tracking revealed that the increase of both E cells and M cells in suspension coculture of E and isogenic M cells is not a consequence of sequential EMT and MET at the single cell level but consistent with mutual E and M cell cooperation [37] which stimulated M cell expansion and persistence of E cells.

### Soluble factors and cell-cell contact mediate cooperation of E and M cells

**Figure 4:**
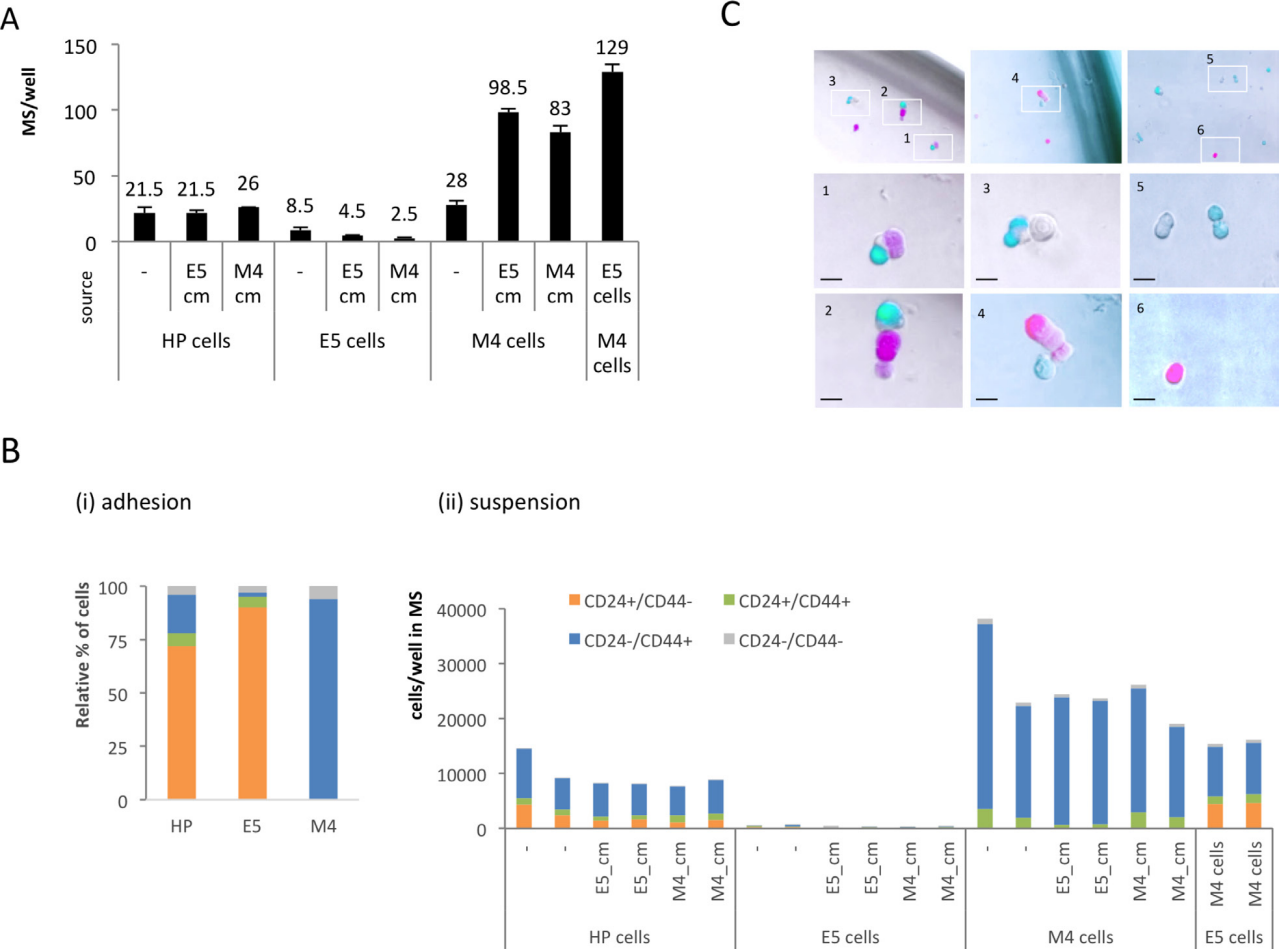
The role of soluble factors and direct cell-cell contact for synergistic effect of E/M coculture in suspension (**A**) Relative numbers of mammospheres from HP, E5 or M4 clone cells after treatment with conditioned medium (CM) of adherent E or M cells or E/M coculture. Average of data performed in duplicates is shown. (**B**) Quantitative flow cytometry of the four different CD24/CD44 subpopulations from (i) in adhesion grown source cells and from (ii) cells found in dissociated mammospheres after mono- and coculture of E and M cells with or without CM. (**C**) Spontaneous heterogeneous aggregates of freshly sorted CMV-driven mCherry-labelled CD24−/CD44+ (M) and CMV-driven GFP-labelled CD24+/CD44− (E) cells after 2 days culture under mammosphere conditions.

We reasoned that this mutual cooperation in suspension of HMLER E and M cells could either be mediated by soluble paracrine factors released by the partner cell type or by direct cell-cell contact. Thus, we cultured E cell lines (E5 and HP cell lines with ∼90% and ∼72% E cells in adhesion, respectively) and pure M cells in the presence of conditioned medium from adherent M4 cells (M4_cm) or from adherent E5 cells (E5_cm) (Figure 4A). Suspension culture of M4 cells with E5_cm led to an increase of total mammospheres and almost resembled the mammosphere increase observed by coculturing E and M cells. This suggested that soluble E factors can mediate the effect of coculture towards increased mammosphere formation and proliferation of M cells. By contrast, there was no stimulatory effect of M4_cm on mammosphere formation of E cells (neither in E5 nor HP cell lines) (Figure 4A).

Consistently, cell-type analysis by CD24 and CD44 phenotyping of E or HP-derived mammospheres cocultured with the conditioned medium M4_cm showed no increase in E cell numbers (Figure 4B). As expected, no CD24+/CD44− E cells were found in dissociated mammospheres of M4 cells cultured with E5_cm or M4_cm, again pointing to the absence of MET and the stably transdifferentiated state represented by the M clones. Thus, soluble paracrine E factors may be able to mediate the cooperation towards increased proliferation of M cells in suspension but do not induce MET, whereas no paracrine M cell factors could support persistence of E cells in suspension (Figure 4B).

Next, we investigated cell-cell contact as possible mechanism for the cooperation through which M cells could confer persistence to E cells. While originally it had been proposed that mammospheres are clonal [38], more recently the picture that mammosphere actually can be aggregates of heterogeneous cell types has gained acceptance [39, 40]. If cell contact with M cells produced E cells persistence, we expected that E and M cells would form heterogeneous aggregates in suspension mammosphere cultures. To test this hypothesis, we transduced HP cells with a vector to constitutively express GFP (P_CMV_-YFP) or mCherry (P_CMV_-mCh), freshly sorted 200 individual mCh+/CD24+/CD44− (E) cells together with 200 individual YFP+/CD24−/CD44+ (M) cells, and cocultured them under mammosphere conditions. We then imaged suspension cultures after two days. Indeed, we observed heterogeneous multicellular mCh+/YFP+ aggregates (Figure 4C), suggesting that E and M cells exhibit affinity to each other even at very low cell densities and that aggregation with M cells may inhibit detachment-induced anoikis of E cells.

### M signatures predict more favorable patient outcomes than E signatures in basal breast cancer

Finally, we examined if recent findings in mouse models of M cells not being MICs [28, 28, 30, 31] and the HMLER cell tracking results presented here in support of the cooperation metastasis model are reflected in clinical data. Since metastasis is linked to poor patient survival our *in vitro* data would suggest that in patients the E signatures predict worse outcomes than M signatures. To test this hypothesis, we analyzed the predictive power of E and M signature expression for relapse free survival (RFS) and overall survival (OS) of up to 1764 or 626 breast tumor patients, respectively, by applying the Kaplan-Meier plotter (KMP) online tool [41, 42].

**Figure 5:**
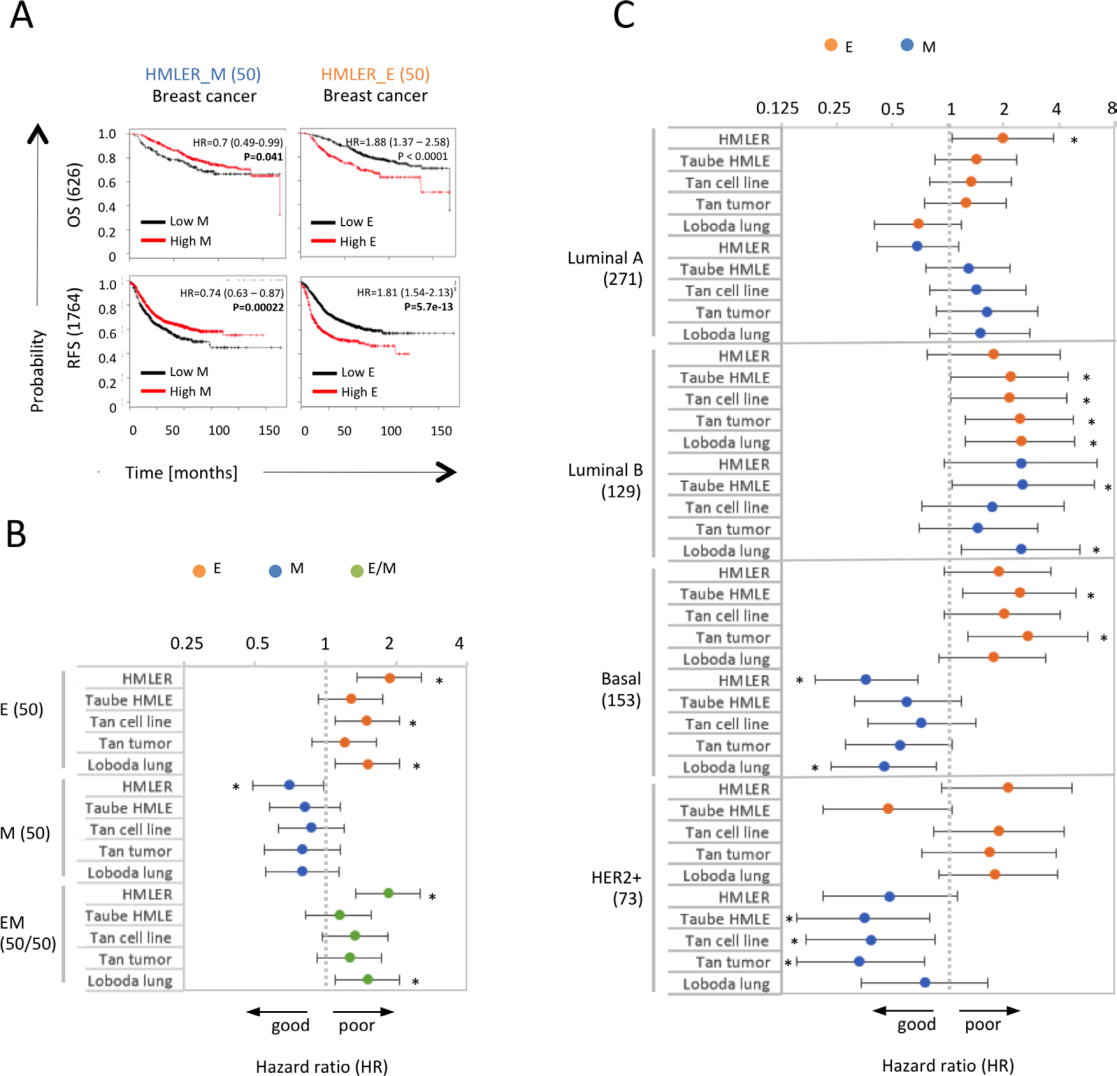
M signatures indicate more favorable patient outcomes than E signatures in basal breast cancer (**A**) Kaplan Meier plots for RFS and OS associated with HMLER_E and HMLER_M signatures in breast cancer. Patient numbers, hazard ratio (HR), 95% confidence intervals, and logrank *p*-values (P) for respective signatures are indicated. (**B**) HR for OS associated with E, M and E/M composite signatures derived from different sources in breast cancer (626 patients). Asterisks indicate signatures predicting OS with *p* values < 0.05. (**C**) HR for OS associated with different E and M signatures in different breast cancer subtypes. Analyzed patient numbers are indicated in brackets.

Using previously defined E- and M-specific HMLER-derived gene expression signatures (Supplementary Figure 4A, Supplementary Table 2, Methods) in the complete KMP breast cancer database, we found that the M_HMLER signature was significantly associated with favorable patient outcomes in terms of OS (HR = 0.7, *p* < 0.05) and RFS (HR = 0.74, *p* < 0.001). By contrast, the corresponding E gene set based on the E_HMLER signature predicted significantly poor outcomes (HR[OS]=1.88, HR[RFS]=1.81, p<0.0001) (Figure 5A). To ensure that these findings were due to particularities of the HMLER-specific gene sets, we rerun the analysis for four additional gene sets for the E and M signatures, derived independently from breast (Taube HMLE, Tan cell lines) and lung (Loboda) cancer cell lines [43, 49, 64] and different tumor types (Tan tumor) [49] (Methods and Supplementary Table 2). These alternative M gene signatures corroborated the above findings and indicated more favorable OS (between HR = 0.8 and 0.87) (Figure 5B), while the alternative E gene sets consistently predicted worse OS (between HR = 1.21 and HR = 1.52). However, the differences between the corresponding E and M signatures for these four additional E and M signature sets had overlapping 95% confidence intervals. Consistent with the reported stemness of the intermediate E/M state [12] the combined presence of E and M signatures also predicted poor outcomes (between HR = 1.14 and HR = 1.85, Figure 5B), resembling the outcome predicted by the E signatures.

By induction of EMT in HMLE cell lines using siCDH1, SNAI1, TWIST, and GSC overexpression, or TGFβ treatment [43] Taube *et al.* had generated five different corresponding E and M signatures (Supplementary Table 2). An older KMP-database version significantly predicted favorable OS and RFS in breast cancer patients for these Taube M signatures alone (between HR = 0.58 and HR = 0.66, *p* < 0.017). Outcomes associated with M signatures were consistently better (between HR = 0.58 and HR = 0.63) than their corresponding E signatures alone (between HR = 0.76 and HR = 1.4) or E signatures in combination with M signatures (between HR = 0.73 and HR = 0.88, Supplementary Figure 4C). Together, these observations are in agreement with the hypothesis of cooperation between E and M cells and of E cells being highly plastic and having potentially MIC character.

To determine in which breast cancer subtype enrichment of the M signatures was associated with better outcomes and induction of complete EMT may possibly even have therapeutic benefit we used the St. Gallen criteria classification of the KMP and divided the tumors in the KMP database into the four canonical intrinsic subtypes: the ER-positive luminal A and luminal B tumor subtypes, the ER-negative basal and the HER2-positive subtypes [41, 44]. Intriguingly, in the basal breast cancer subtype we observed that all five M signatures consistently predicted more favorable outcomes (between HR = 0.36 and HR = 0.72), while their corresponding E signatures predicted poor outcomes (between HR = 1.75 and HR = 2.7). These differences between E and M signatures were significant within a 95% confidence interval for four out of the five signatures tested (Figure 5C). The same trend of E signatures predicting worse outcomes, albeit not within 95% intervals, was observed for the five different Taube EMT signatures in the basal subtype in an older database (Supplementary Figure 4C). In none of the other breast cancer subtypes was a consistent difference for outcomes between tumors with predominant E and M signatures observed (Figure 5C); in the luminal A and B subtypes both E and M signatures were associated with poor OS, while in the HER2+ subtype four of the five tested E signatures (between HR = 1.67 and HR = 2.1) were also associated with worse outcomes than M signatures (between HR = 0.33 and HR = 0.75), which was significant within a 95% confidence interval for two of the five tested E and M signature pairs.

In conclusion, patient data show that E signatures are mostly associated with worse outcome than M signatures in breast cancer, thus supporting the hypothesis that E cells rather than M cells represent the MICs in breast cancer, particularly in basal breast cancer.

## DISCUSSION

Most life-threatening macrometastases in breast cancer are epithelial and resemble the primary tumors. However, it is still unclear how anoikis-prone E cells reach distant metastatic sites after detachment, and whether M or E represent the MICs that give rise to the E metastases. Using the basal-like breast cancer cell line HMLER, we present here *in vitro* evidence that MICs may originate from cells in the epithelial state and that mutual cooperation with M cells can support the survival and persistence of a pre-existing CD24+/CD44– (E) subpopulation and lead to E cell proliferation in suspension culture. We further provide evidence that M cells can reside in the stable (irreversible) M transdifferentiated state such that they are unlikely to undergo MET but rather function to aid in stabilizing the E population by suppressing their anoikis and plasticity, i.e., their inherent potential to undergo EMT. Thus, our data are consistent with a series of recent mouse studies demonstrating that M cells do not give rise to metastasis by MET [6, 30, 31, 33, 34]. Consistent with our *in vitro* data, clinical data show that M signatures predict better outcomes in breast cancer patients than E signatures, particularly in basal breast tumors. Together, these data support the cooperation metastasis model in which E cells are the MICs.

In our previous study we had determined that stemness was associated with the intermediate E/M state for most breast cancer subtypes as well as for the HMLER E and M cell lines. However, it remained unclear whether stemness depended upon individual stem-like hybrid E/M cells or on the mixture of M and E cells which would suggest E-M cooperation [12]. By analyzing individual clonal E and M populations alone as well as E and M mixtures, and reporter cell lines, our present data suggest the existence of two different types of hybrid E/M cells: (i) hybrid E/M cells derived from M cells which were instable and underwent EMT when allowed to readhere, and (ii) hybrid E/M cells derived from E cells which were stable and found in the majority of tested E population (Figure 2C). These observations reinforced our previous notion that HMLER E cells display much more plasticity along the E-M axis than M cells, and that E cells are more likely to give rise to heterogeneous E and M signatures in HMLER cell populations with stemness properties [12]. We conclude that at least in HMLER cells mainly E cells have the potential to give rise to cooperating E and M populations. If that holds true for breast cancer in general, then pre-existing and residual E cells from the original tumor would be necessary to produce at a metastatic site the same epithelial heterogeneity as in the primary tumor. The observation that in breast cancer patients in general, and particularly in the basal subtypes, better survival correlates with M signatures supports this notion, and is in agreement with findings of other reports that used different and smaller databases [43, 49].

Our results on the absence of complete MET at the single cell level in HMLER cells stand in sharp contrast to the popular assumption that metastasis is caused by cellular plasticity of individual highly aggressive cancer stem cells [13, 45] that undergo EMT, thereby generating more aggressive cell types [4, 5]. According to this perspective, M cells resulting from EMT are considered the MICs and will have to eventually undergo MET, in accordance to the sequential metastasis model [17, 46]. Our results suggest that pre-existing E cells acting as MICs, generate the adherent E population after replating following a suspension phase and represent the majority of cells in our HMLER model; however, we cannot exclude the occasional existence of M MICs and of spontaneous METs that may be triggered by rare mutations. In support of our findings, we observed that EMT was nearly inevitable for individual E HMLER cells once detached from the adhesion state, which may also apply to metastatic E cells leaving the primary tumor (Figures 1, 2). Furthermore, EMT even occurred in M clones that had transiently undergone a partial MET (Figure 1B). Thus, any spontaneous partial MET would be overridden by the default EMT process upon detachment and readhesion. Finally, although our data suggest that MET at the *single cell level* may be unlikely, the migratory and less adherent nature of M cells in proliferating mixed populations may lead to an enrichment of E cells and an apparent (population level) MET in certain metastatic solid tumors.

A few but important experimental differences to earlier studies [4, 13, 45] may underlie the discrepancy between previous reports and our results. First, we have not only used a single cell line but a series of single cell-derived clones as well as stable reporter cell lines, which has enabled us to identify the individual E and M cell fate (Figure 1B). Second, previous studies [4, 5, 45] used mixed E and M populations for sorting. However, mixed cell populations (as shown for HMLER cells) may still contain two types of CD24−/CD44+ (M) cells, pure M cells originated from completed EMT, as well as stem-like hybrid E/M cells which may have been directly and recently derived from the highly plastic E cells [12]. To overcome this bias, we used here clonal populations or stable M reporter cell lines that had undergone complete EMT and only contained pure M cells as determined by single cell analysis (Figure 2C). Third, to track cell fates we cultured cells under suspension mammosphere conditions that allowed nearly clonal expansion that mimic the metastatic process that is thought to start from few cells. By contrast in other studies [4, 5, 45] large numbers of E versus M cells were sorted directly into highly charged 2D adhesion plastic dishes which favors an apicobasal polarity and adhesion as is needed for the E phenotype, potentially preventing complete EMT of “contaminating” E cells. Along the same lines, it has been suspected that 2D adhesion cultures do not accurately replicate cancer cell behavior in patients, and that 3D or mammosphere suspension cultures better reflect tumor heterogeneity [38, 47, 48]. Thus, the controversial experimental results may be explained by our experimental conditions that model metastasis by detachment of tumor cells, a prolonged suspension culture phase, and readhesion as opposed to experimental conditions that relied on cells exclusively cultured in adhesion.

Given that we did not observe plasticity in the sense of MET *in vitro,* dominance of EMT upon detachment and readhesion as well as the significant association of M signatures with favorable patient survival in basal tumors, it could be assumed that complete EMT in individual breast-derived cells may represent an irreversible *transdifferentiation* into a non-tumorigenic state and associated tumor microenvironment, as opposed to a reversible *transition* into aggressive cells. The tendency of the epithelial HMLER cell lines to undergo transdifferentiation towards the M state upon detachment may thus explain their low tumorigenicity and absence of metastasis in mouse models [49]. Even upon normal passaging for more than 15 passages, HMLER cells tended to spontaneously undergo EMT (Supplementary Figure 1A), and thus EMT of E populations and tumors may be inevitable during development. Indeed, a combination of experimental data and mathematical modelling demonstrated that e.g. the transcription factor GRHL2 stabilizes the intermediate E/M state and predicts poor outcomes in breast, kidney, lung, and liver cancers [50].

Mechanistically, we present evidence at the cellular level that cooperation is not mainly mediated by soluble factors but also by direct cell-cell contact of pre-existing HMLER E cells and stably transdifferentiated M cells (Figure 4C). Thus direct E-M cell contact may prevent EMT of E cells. This is in contrast to studies showing that mammospheres are clonal [38], but consistent with several recent studies demonstrating non-clonality of mammospheres [40]. Our results are also in agreement with observations that circulating tumor cell (CTC) clusters are substantially more tumorigenic than individual CTCs [51], and that fresh patient-derived tumors are best propagated in mice as clusters. More studies are needed to understand if E and M cell clustering can prevent EMT in primary tumors, and which molecular mechanisms mediate the clustering. Furthermore, our data on cooperation of E cells with the transdifferentiated M cells support studies suggesting that clustering with stromal cells of the tumor microenvironment, such as mesenchymal stem cells [52, 53] or fibroblasts [54, 55] can promote outgrowth of metastatic breast cancer cells.

## MATERIALS AND METHODS

### Cell culture

HMLER cells are primary human mammary epithelial cells (HMECs) transformed to carcinoma cells through the introduction of SV40 large T and Ras oncogene [35]. HMLER cells were kindly provided by Robert A. Weinberg (Whitehead Institute). Parental HMLER (HP) cells, single cell-derived epithelial (E1 to E6) and mesenchymal HMLER clones (M1 to M5) were described previously [12]. All HMLER-derived cell lines were passaged in adhesion under serum-free culture conditions in a 1:1 mix DMEM (Life Technologies)/MEGM (Lonza). Conditioned medium (CM) was harvested from adherent nearly confluent E5 and M4 cells and separated from suspended cells by centrifugation.

Mammospheres (MS) were generated as described earlier [38]. Briefly, adherent cells were trypsinized, and (unless otherwise stated) cell numbers of 10,000 to 20,000 cells/ml were either flow cytometry-sorted or pipetted into ultra-low attachment (ULA) plates (96 well plates, 100ul volume, Corning). Mammospheres were cultured for 2–3 weeks in DMEM/MEGM supplemented with 1× B27 (Life Technologies) and 1% methylcellulose (viscosity 4,000cP, Sigma) in biological duplicates. E/M cocultures were always generated from equal numbers of E and M cells, and compared to equal total numbers of E or M monocultured cells. Mammospheres were counted by eye with a 4x or 10x objective. For replating mammospheres to adhesion cultures, 50% of dissociated non-stained mammosphere suspension cultures was transferred into normal adhesion culture conditions and analyzed by quantitative flow cytometry after one week. For gene expression arrays replated cultures were analyzed after 6 hours to 10 days (for HP cells and M clones) and after 2–3 weeks (E clones) as indicated in the text.

### Sorting and single cell qPCR analysis

Single cell analysis was performed as described before [12]. Briefly, live single cells of equal FSC/SSC morphologies within the middle of the respective fluorescent gates were sorted directly into 96 well-plates. Wells contained Taqman primers and Cells Direct One-step RT-PCR and pre-amplification mix (Life Technologies) followed by RT and 18 cycles of preamplification in a GeneAmp PCR System 9700 instrument (Applied Biosystems) according to the Fluidigm single cell protocol. Gene expression was analyzed on a BioMark instrument using 48.48 Dynamic Array IFC chips (Fluidigm) and Taqman primers (Applied Biosystems).

Single cell data were analyzed similar as described previously [12]. Briefly, measured Ct values were transformed into linear expression values according to Fluidigm's single cell application protocol. Expression data for every E gene was normalized to the value of the maximum measured for the same gene in a simultaneously analyzed 100-cell samples of adherent E cells (HP and E4 cells) and divided by 100, while M genes were normalized for maximum measured 100-cell value in adherent M5 cells. To plot single cell gene expression values into the E/M state space, normalized expression values per single cell were averaged for 9 E genes (CDH1, EPCAM, KRT5, LCN2, S100A8, S100P, SLPI, TP63, TNFSF10/TRAIL) and 11 M genes (ABCA6, AR, CDH2, DCN, FN1, PCOLCE, SNAI1, VIM, WNT5A, ZEB1, ZEB2).

### Gene expression arrays and principal component analysis

RNA of cells grown in adhesion was isolated by lysing PBS-washed cells by addition of Trizol (Life Technologies) to the adherent cells in the dish, as detaching of cells before Trizol resulted in substantial RNA degradation. RNA was purified according to the manufacturer's instructions. Gene expression analysis was performed essentially as described [12]. Briefly, 50 ng to 100 ng RNA was labeled using the one color Low Input Quick Amp Labeling Kit (Agilent), and labeled probes were run on Human 4 × 44 K Microarrays (Agilent) according to the manufacturer's instructions. Data were analyzed with Genedata Analyst 7.0 (Genedata, Basel, Switzerland). Data normalization was performed using central tendency followed by relative normalization. Principal Component Analysis (PCA) of gene expression array data was performed using the indicated 150 most E- and M-specific signatures by using a covariance matrix in GeneData Analyst 7.0.

The complete microarray dataset shown in Figure 1 has been deposited in NCBI's Gene Expression Omnibus and is accessible through GEO accession numbers GSE66527 (adherent cells and mammospheres) and GSE70279 (replated cells). RNA of adhesion cultures was derived from five different freezing passages of HP cells, five HMLER M clones and six different HMLER E clones (biological duplicates). RNA of readhesion cultures was derived from HP cells (10 biological replicates from four different passages), and three different E clones and M clones (biological duplicates).

### Staining and flow cytometry analysis

Single cell suspensions of trypsinized adherent growing cells or dissociated mammospheres were stained with antibodies (αCD24-PE: clone ML5, αCD44-FITC or αCD44-APC: clone G44–26, BD-Biosciences) diluted 1:25 in DMEM supplemented with 2% FBS and 25 mM HEPES. Flow cytometry data acquisition was performed on a FACSAria II SORP (Becton Dickinson) and analyzed with FlowJo software (Tree Star, vX.0.6). To determine cutoffs for the CD24/CD44 quadrants for any cell line, simultaneously stained heterogeneous HP cells (expanded in adhesion) were included as a reference. Relative cell numbers in mammosphere cultures within an experiment were determined by ‘quantitative flow cytometry’ assessing cell numbers in the same volumes and same time. All data shown are representative of at least two experiments performed in biological replicates.

### Generation of fluorescent E and M reporter cell lines

For tracking origin of E and M cells, we generated a lentiviral dual fluorescent CDH1-promoter reporter vector (pLenti6-P_CDH1_-mCherry_P_SV40_-YFP). The 8.6kb long CDH1-promoter reporter vector could track CDH1 transcriptional activation by driving fluorescent mCherry expression and simultaneously showed presence of the promoter reporter construct itself by using the constitutively active SV40 promoter driving expression of yellow fluorescent protein (YFP). Using the commercially available pLenti6/V5-D-TOPO vector (Thermo Fisher) as a base, we exchanged the CMV promoter with the human CDH1 promoter (P_CDH1_). A sequence of 1122 bp of the human CDH1 promoter region (-1178 through -57 upstream of the initiator methionine) was PCR amplified using 293T cell genomic DNA using the forward primer (5′ agatcagcctcggcaacatagtg 3′) and reverse primer (5′ gctggagcgggctggagtctga 3′). The CDH1 promoter drives the expression of a 711 bp mCherry open reading frame [60]. The blasticidin ORF of the original vector pLenti6/V5-D-TOPO was replaced by the 720 bp long YFP ORF [61], so that YFP is constitutively expressed by the P_SV40_ promoter of the original vector.

Transduction of heterogeneous HP cells was performed using the Virapower Lentiviral Expression Systems kit (Life Technologies). Briefly, the CDH1-mCherry reporter construct together with pLP1, pLP2, and pLP/VSVG was transfected into 293FT cells. HP cells were then transduced with the virus-containing supernatant, expanded, and after cell sorting the two YFP-positive cell lines (mCherry-positive E_YFP+ and mCherry-negative M_YFP+) were derived and expanded. Single cell qPCR data (Figure 2) and cytometry data (Figure 3) shown are representative for two independently derived E_YFP+ and M_YFP+ cell lines.

### Microscopy

Cells were imaged on a DM IL LED instrument (Leica) with a DFC345 FX camera or on a DeltaVision Core instrument (GE Healthcare).

### Kaplan-Meier survival analysis

The ‘Kaplan-Meier Plotter’ (KMP) online tool (kmplot.com) was used with either the 2017 breast cancer dataset (1764 and 626 patients for ‘relapse-free survival’ (RFS) and ‘overall survival’ (OS), respectively). According to the KMP tool breast cancer (all BC subtypes) is classified into four clinically relevant intrinsic subtypes based on the St. Gallen criteria using bimodal expression of estrogen receptor (ER, *ESR*), expression of HER2 (*HER2*), and the proliferation marker Ki67 (*MKI67*) into luminal A (ESR+, HER2-, MKI67low), luminal B (ESR+,HER2-, MKI67high and ESR+, HER2+), basal (ESR-, HER2-) and HER2+ (ESR-, HER2+) patients [41, 42]. Hazard ratio (‘HR’, within 95% confidence intervals) and logrank *p*-values (Cox’ proportional hazard ratio analysis) for survival of breast cancer patients [41] was determined by the mean expression of the respective gene signatures (Supplementary Tables 1 and 2) [12, 43, 62, 63]. The ‘autoselect best cutoff’ option [64] was used for subdividing gene expression in primary tumors by median, quartile or tertile expression, and ‘JetSet best probe set’ [44] option was used to assess HR, 95% confidence intervals and logrank *p*-values for patient survival.

### Gene signatures

All E and M gene signatures were derived by comparing epithelial versus mesenchymal cell lines cultured *in vitro* or in mice, and contained the 24 (Supplementary Table 1), 50 (Supplementary Table 2) or 150 [12] most differentially expressed genes. E_HMLER and M_HMLER signatures were defined from HMLER E and M clones as described before [12]. The Taube HMLE E and M signatures were derived from HMLE cells induced to undergo EMT by transduction with siCDH1, SNAI1, TWIST, GSC, or treatment with TGFβ, or from the overlap respective overlap (Taube HMLE) [43]. E_Tan and M_Tan signatures were based on breast cancer cell lines or tumors [63], and E_Loboda and M_Loboda signatures were derived from lung tumors [62].

## CONCLUSIONS

To our knowledge the intriguing consequence of complete EMT irreversibly converting E to M cells, hence eliminating MICs has not been explored yet. It is generally assumed that tumors become gradually more aggressive over time accumulating conversion into M cells. Hence, much effort went into exploring the inhibition of EMT and inducing MET as a therapeutic strategy [56, 57]. This may be detrimental in view of the findings presented here where enrichment for M signatures are associated with better outcomes and E signatures with worse outcomes in breast cancer. It is noteworthy that basal tumors are considered more mesenchymal tumors than luminal tumors. In support of the new notion that the M state is less aggressive, and thus, that EMT does not necessarily result in more aggressive tumors, basal breast cancer patients have a indeed a higher pathological response rate and better long term survival than luminal breast cancer patients, albeit worse short term outcomes [58, 59].

Our *in vitro* and patient analysis suggest that tumor-specific therapeutic induction of complete EMT may have a long lasting clinical benefit in basal (triple-negative) breast cancer patients due to a potential irreversibility of EMT of individual cells and corresponding inhibition of epithelial metastases. Particularly patients with basal ER-negative breast tumors have an unmet clinical need due to absence of targeted therapies for this subtype. Given that M signatures are associated with favorable outcomes these patients may benefit from EMT induction.

## SUPPLEMENTARY MATERIALS FIGURES AND TABLES







## Abbreviations

BC: breast cancer
CM: conditioned medium
CSC: cancer stem cells
E: epithelial
HP: parental HMLER cells
HR: hazard ratio
EMT: epithelial-to-mesenchymal transition
KMP: Kaplan-Meier Plotter
M: mesenchymal
MET: mesenchymal-to-epithelial transition
MS: mammospheres
OS: overall survival
PCA: principal component analysis
RFS: relapse-free survival
YFP: yellow fluorescent protein
