# Supplementary material for: Loss of inter-cellular cooperation by complete epithelial-mesenchymal transition supports favorable outcomes in basal breast cancer patients
Source: Oncotarget. 2018 Apr 13;9(28):20018–33. doi: 10.18632/oncotarget.25034 (PMC5929443; doi:10.18632/oncotarget.25034)
Supplement: Supplementary file 1 [file oncotarget-09-20018-s001.pdf]

Loss of inter-cellular cooperation by complete epithelial-mesenchymal transition supports favorable outcomes in basal breast cancer patients

SUPPLEMENTARY MATERIALS

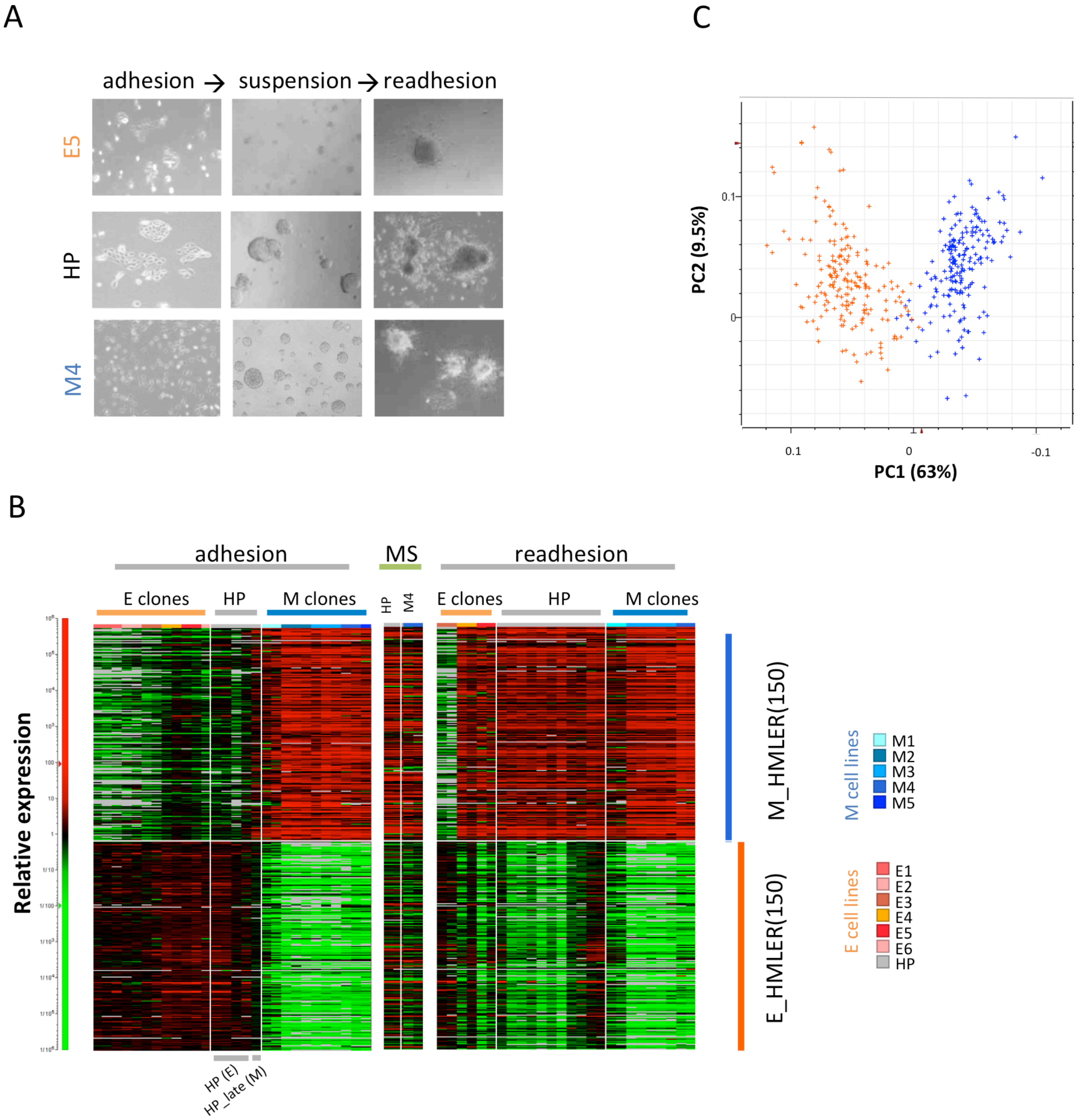

Supplementary Figure 1: Enrichment of M signatures upon suspension culture and replating. (**A**) Micrographs with representative morphology for HMLER cell lines. (**B**) EMT is inevitable during suspension culture and replating of HMLER cells. E and M_HMLER (150) signature expression in HMLER E and M clones, and HP cells, shown at different passages in adhesion, in suspension mammospheres (MS) and after readhesion. Note a spontaneous enrichment for M genes in adherent HP cells in late passages (HP_late, after about 15 passages) versus earlier passages (HP). (**C**) PCA loading plot for 150 E and 150 M genes from experiment in Figure 1A, 1B.

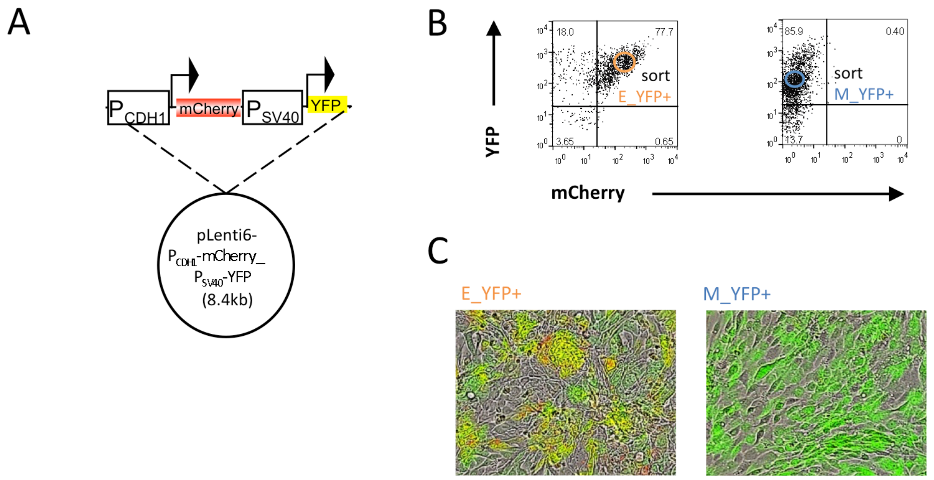

Supplementary Figure 2: A dual-fluorescent lentiviral CDH1 promoter reporter vector. (**A**) Lentiviral dual fluorescent CDH1-promoter reporter vector to monitor epithelial CDH1-driven mCherry and constitutive SV40-driven YFP gene expression. (**B**) FACS analysis of E_YFP+ (YFP+/mCherry+) and M_YFP+ (YFP+/mCherry−) cell lines. Indicated gates were used to sort E and M cells from E_YFP+ and M_YFP+ cell lines, respectively, for experiments shown in Figure 3. (**C**) Stable cell lines E_YFP+ and M_YFP+, ~40× magnification. E/M heterogeneity of E_YFP+ cells is visible by green and red fluorescence relative to homogenously green M_YFP+ cells.

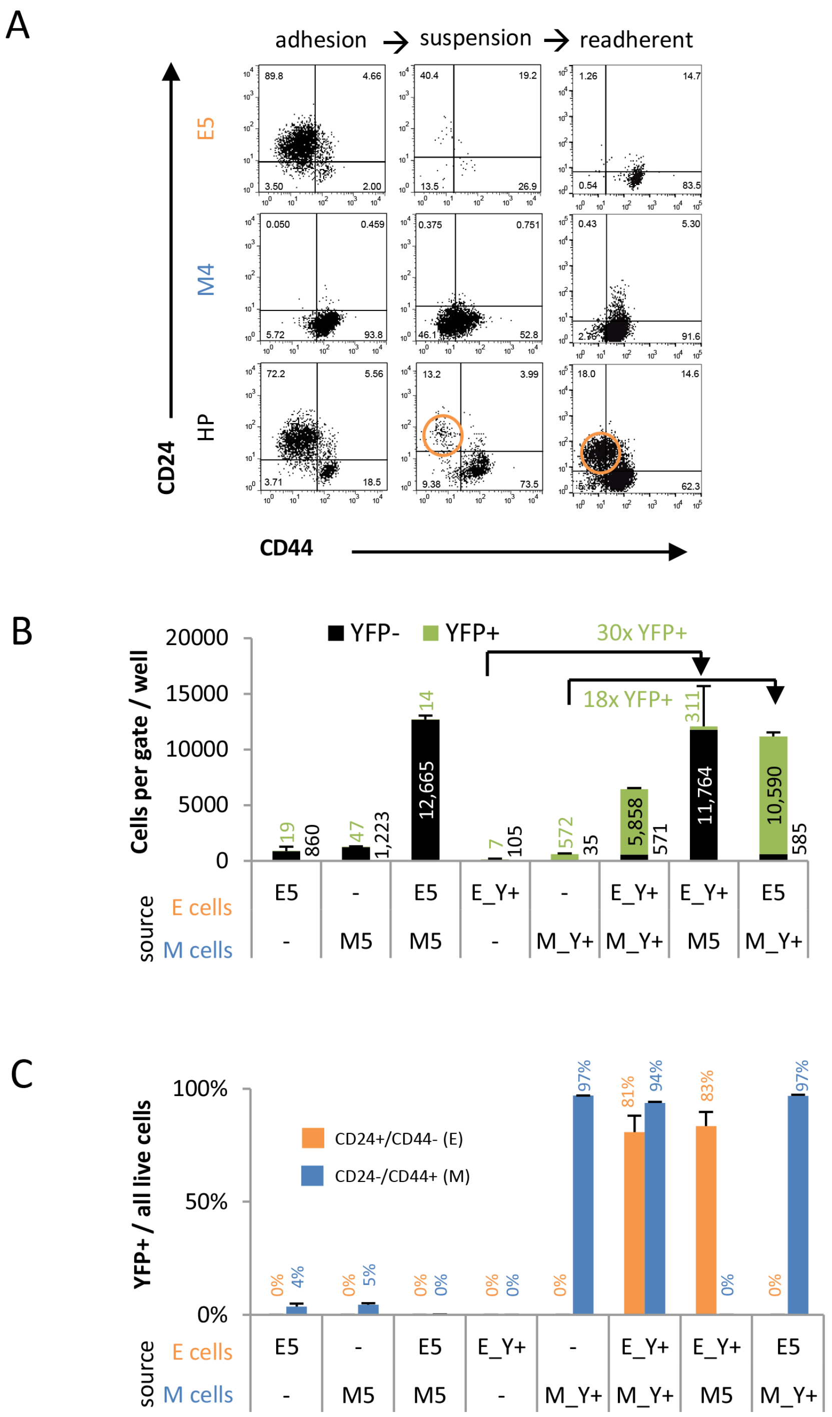

Supplementary Figure 3: Tracking origin of surviving co-cultured E and M cells in suspension. (**A**) Quantitative CD24/CD44 flow cytometry profiles of E5, M4, and HP cells grown in adhesion, suspension (16,000 cells/100 ul), and suspension followed by readhesion. (**B**) Quantitative flow cytometry live cell counts from E and M cell cocultures. Total number of live cells per well of biological duplicates in YFP+ and YFP− gates are plotted. Fold increase of YFP+ cells (green) in E/M cocultures versus respective E or M monocultures is indicated. (**C**) From same experiment as shown in B and Figure 3B, 3C the fraction of YFP+ cells relative to all live cells in E (CD24+/CD44−) or M (CD24−/CD44+) quadrants is shown as orange or blue bars, respectively.

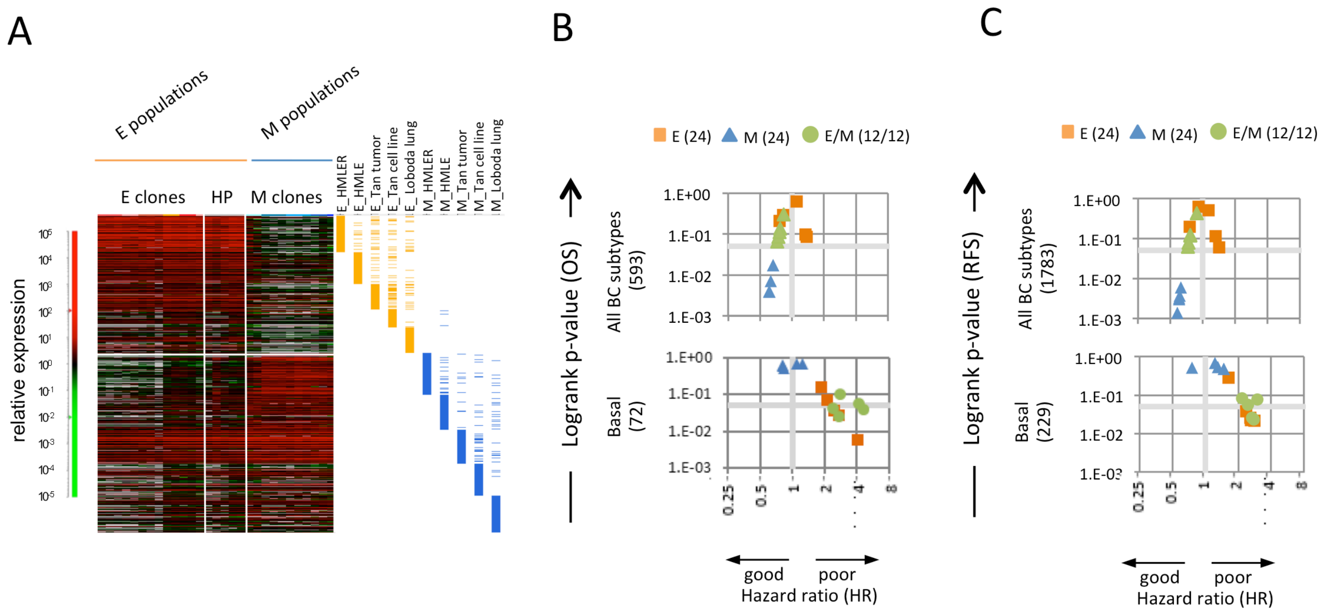

Supplementary Figure 4: Expression of different E and M signatures in adherent HMLER cell lines and their predictive meaning for survival of breast cancer patients. (**A**) Overlap of expression of different 60 genes comprising E(60) and M(60) signatures in arrays from HP cells and HMLER E and M clones displayed in a heatmap. (**B**) HR for overall survival versus logrank *p*-value plots associated with expression of five different overlapping breast-specific E and M signatures (24 genes) from Taube et al in BC subtypes together or in basal breast cancer patients (database: Kaplan–Meier-Plotter version 2010). Analyzed patient numbers given in brackets. (**C**) HR for relapse-free survival versus logrank *p*-value plots associated with expression of five different Taube-derived E and M signatures.

Supplementary Table 1: E- and M-specific signatures (24 genes). See Supplementary_Table_1

Supplementary Table 2: E- and M-specific signatures (50 genes). See Supplementary_Table_2
