# Supplementary material for: Loss of inter-cellular cooperation by complete epithelial-mesenchymal transition supports favorable outcomes in basal breast cancer patients
Source: Oncotarget. 2018 Apr 13;9(28):20018–33. doi: 10.18632/oncotarget.25034 (PMC5929443; doi:10.18632/oncotarget.25034)
Supplement: Supplementary file 2 [file oncotarget-09-20018-s002.docx]

**Supplementary Table 1: E- and M-specific signatures (24 genes).**

| **Reference** | **Grosse-Wilde et al, 2015** | |  |  | **Taube et al, 2010** | |  | **Taube et al, 2010** | |  | **Taube et al, 2010** | |  | **Taube et al, 2010** | |  | **Taube et al, 2010** | |  |  |  |
| --- | --- | --- | --- | --- | --- | --- | --- | --- | --- | --- | --- | --- | --- | --- | --- | --- | --- | --- | --- | --- | --- |
| Parents | HMLER | | |  | HMLE | |  | HMLE |  |  | HMLE |  |  | HMLE |  |  | HMLE | |  |  |  |
| EMT induction | spontaneous clones | | |  | siCDH1 overexpression | |  | TWIST |  |  | GSC |  |  | SNAI1 |  |  | TGFβ treatment | |  |  |  |
|  |  |  | |  |  |  |  |  |  |  |  |  |  |  |  |  |  |  |  |  |  |
| **Ranking** | **E_HMLER (24)** | **M_HMLER (24)** | |  | **E_siCDH1 (24)** | **M_siCDH1 (24)** |  | **E_TWIST (24)** | **M_TWIST (24)** |  | **E_GSC (24)** | **M_GSC (24)** |  | **E_SNAI1 (24)** | **M_SNAI1 (24)** |  | **E_TGFβ (24)** | **M_TGFβ (24)** |  |  |  |
| 1 | KRT6B | NOV | |  | SERPINB2 | RGL1 |  | SAA1 | ROR1 |  | FGFBP1 | PPAP2B |  | EPCAM | ROR1 |  | SPRR1B | TBX3 |  |  |  |
| 2 | KRT6A | STXBP6 | |  | KLK10 | BIN1 |  | UCHL1 | NR2F1 |  | EPCAM | C10ORF56 |  | SERPINB2 | MYL9 |  | FGFR3 | RGS4 |  |  |  |
| 3 | FGFBP1 | GPM6B | |  | SPRR1B | MME |  | SPRR1A | DNAJB4 |  | S100A14 | PMP22 |  | SPRR1A | SPOCK1 |  | SPRR1A | SRGN |  |  |  |
| 4 | KRT5 | GREM1 | |  | S100A8 | DLC1 |  | FGFR3 | MME |  | KRT5 | TRAM2 |  | SAA1 | WNT5A |  | SERPINB2 | PVRL3 |  |  |  |
| 5 | KRT6E | HHIP | |  | SLPI | NEBL |  | SERPINB2 | SPOCK1 |  | SPRR1B | ROR1 |  | ANXA3 | C5ORF13 |  | KLK10 | XYLT1 |  |  |  |
| 6 | KLK10 | NR2F1 | |  | SPRR1A | PRR16 |  | KLK10 | FBLN1 |  | CDH3 | PTX3 |  | UCHL1 | NEBL |  | EPCAM | ROR1 |  |  |  |
| 7 | S100A14 | GGTLA1 | |  | KLK7 | SPOCK1 |  | CCND2 | PMP22 |  | KRT17 | PVRL3 |  | RAB25 | PTX3 |  | UCHL1 | OLFML3 |  |  |  |
| 8 | EPCAM | COL6A2 | |  | ALDH1A3 | LTBP1 |  | SLPI | RGS4 |  | KRT6B | CTGF |  | CCND2 | TNFAIP6 |  | RAB25 | RGL1 |  |  |  |
| 9 | SPINT2 | DCN | |  | KRT15 | NR2F1 |  | CXADR | PCOLCE |  | ANXA8 | SRGN |  | SLPI | NR2F1 |  | RBM35A | HAS2 |  |  |  |
| 10 | KLK8 | PTX3 | |  | EPCAM | SRGN |  | CAMK2B | COL5A2 |  | ANXA8L1 | ECM1 |  | TMEM30B | PRRX1 |  | SLPI | MMP2 |  |  |  |
| 11 | SLPI | SNED1 | |  | FST | FBN1 |  | RBM35B | PRRX1 |  | LOC728113 | SEMA5A |  | RBM35B | OLFML3 |  | CXADR | WNT5A |  |  |  |
| 12 | LAD1 | SULT1B1 | |  | PI3 | PRRX1 |  | CA9 | FBLN5 |  | ZBED2 | WNT5A |  | FGFR3 | FBN1 |  | FST | COL5A2 |  |  |  |
| 13 | CDH1 | DKFZP586H2123 | |  | ANXA3 | C5ORF13 |  | RAB25 | MYL9 |  | FGFR3 | IGFBP4 |  | NMU | COL5A2 |  | LGALS7 | COL3A1 |  |  |  |
| 14 | KLK5 | C5ORF13 | |  | KLK5 | RGS4 |  | KLK7 | OLFML3 |  | RBM35A | IGFBP3 |  | FLJ12684 | RGS4 |  | KLK7 | CDH2 |  |  |  |
| 15 | SPRR1B | ITGBL1 | |  | TMEM30B | POSTN |  | S100A7 | FBN1 |  | LEPREL1 | PRR16 |  | KLK10 | PCOLCE |  | S100A7 | COL1A2 |  |  |  |
| 16 | TP73L | LTBP1 | |  | RBM35A | ENPP2 |  | IL18 | WNT5A |  | SAA1 | FBN1 |  | PLS1 | GREM1 |  | TMEM30B | PRRX1 |  |  |  |
| 17 | LAMA3 | PCOLCE | |  | SAA1 | CDH2 |  | KRT17 | POSTN |  | TMEM30B | GREM1 |  | IL18 | FBLN5 |  | EVA1 | TNFAIP6 |  |  |  |
| 18 | COL17A1 | FAM20A | |  | RAB25 | PTX3 |  | HBEGF | SRGN |  | GJB3 | COL5A2 |  | CKMT1A | SRGN |  | KRT15 | CDH11 |  |  |  |
| 19 | FST | TFPI | |  | FGFBP1 | NID1 |  | RNF128 | GREM1 |  | UCHL1 | COL1A2 |  | CKMT1B | ENPP2 |  | CA2 | FBLN5 |  |  |  |
| 20 | ALDH1A3 | COL5A2 | |  | IL1B | DCN |  | HOOK1 | NID1 |  | TRIM29 | NR2F1 |  | SPRR1B | NID1 |  | S100A14 | FBN1 |  |  |  |
| 21 | SFRP1 | COL1A1 | |  | S100A7 | COL1A2 |  | KLK5 | ENPP2 |  | SERPINB2 | POSTN |  | CA9 | COL1A2 |  | CA9 | DCN |  |  |  |
| 22 | KRT14 | GNG11 | |  | FGFR3 | COL3A1 |  | TMEM30B | COL3A1 |  | SPRR1A | NID1 |  | RBM35A | POSTN |  | IL18 | SPOCK1 |  |  |  |
| 23 | IL1B | COL6A1 | |  | SERPINB1 | GREM1 |  | ARTN | COL1A2 |  | CKMT1A | COL3A1 |  | CXADR | DCN |  | IGFBP2 | POSTN |  |  |  |
| 24 | FXYD3 | FBLN5 | |  | C1ORF116 | FBLN5 |  | FLJ12684 | DCN |  | CKMT1B | DCN |  | ALDH1A3 | COL3A1 |  | ARHGAP8 | GREM1 |  |  |  |

24 E and M-specific genes signatures were derived from the indicated publications, and ranked according to their expression with 1 being most highly expressed relative to the population with the opposite morphology.
