# Supplementary material for: Loss of inter-cellular cooperation by complete epithelial-mesenchymal transition supports favorable outcomes in basal breast cancer patients
Source: Oncotarget. 2018 Apr 13;9(28):20018–33. doi: 10.18632/oncotarget.25034 (PMC5929443; doi:10.18632/oncotarget.25034)
Supplement: Supplementary file 3 [file oncotarget-09-20018-s003.docx]

**Supplementary Table 2: E- and M-specific signatures (50 genes).**

|  |  |  |  |  |  |  |  |  |  |  |  |  | |  | |  |
| --- | --- | --- | --- | --- | --- | --- | --- | --- | --- | --- | --- | --- | --- | --- | --- | --- |
| **Grosse-Wilde et al, 2015** | |  | **Taube et al, 2010** | |  | **Tan et al, 2014** | |  | **Tan et al, 2014** | |  | | **Loboda et al, 2011** | | | |
| **E_HMLER (60)** | **M_HMLER (60)** |  | **E_HMLE (60)** | **M_HMLE (60)** |  | **E_Tan Cell line (60)** | **M_Tan Cell line (48)** |  | **E_Tan tumors (60)** | **M_Tan tumors (60)** |  | | **E_Loboda lung (60)** | | **M_Loboda lung (60)** | |
| KRT6B | FBLN5 |  | C20ORF19 | FBLN5 |  | CDH1 | VIM |  | KRT19 | GAS1 |  | | ACPP | | ADAM23 | |
| KRT6A | COL6A1 |  | SNX10 | GREM1 |  | AGR2 | ZEB1 |  | AGR2 | CXCL12 |  | | AGR3 | | ADAMTS1 | |
| FGFBP1 | GNG11 |  | TP73L | COL3A1 |  | EPCAM | EMP3 |  | RAB25 | ZEB1 |  | | ALDH3B2 | | AFF3 | |
| **KRT5** | COL1A1 |  | KCNK1 | COL1A2 |  | KRT19 | SACS |  | CDH1 | GLYR1 |  | | ANK3 | | AK5 | |
| KRT6E | COL5A2 |  | BDKRB2 | DCN |  | RAB25 | AXL |  | ERBB3 | FHL1 |  | | ANKRD22 | | AKAP12 | |
| KLK10 | TFPI |  | ANXA8 | CDH2 |  | TACSTD2 | LOXL2 |  | FXYD3 | FERMT2 |  | | ANXA9 | | ALPK2 | |
| S100A14 | FAM20A |  | ANXA8L1 | ENPP2 |  | S100P | SPARC |  | SLC44A4 | C1S |  | | AP1M2 | | ANGPTL2 | |
| **EPCAM** | **PCOLCE** |  | LOC728113 | POSTN |  | CEACAM6 | FHL1 |  | S100P | FYN |  | | AQP3 | | ANKRD1 | |
| SPINT2 | LTBP1 |  | RHBDF2 | RGS4 |  | GALNT3 | FERMT2 |  | SCNN1A | WIPF1 |  | | ARHGAP8 | | ANTXR1 | |
| KLK8 | ITGBL1 |  | LOC653562 | C5ORF13 |  | FXYD3 | TUBA1A |  | GALNT3 | CYP1B1 |  | | ARHGDIB | | ANXA6 | |
| **SLPI** | C5ORF13 |  | SLC6A10P | PRRX1 |  | SPINT2 | TMEM158 |  | PRSS8 | SERPING1 |  | | ATAD4 | | AOX1 | |
| LAD1 | DKFZP586H2123 |  | SLC6A8 | FBN1 |  | TMEM30B | CALD1 |  | ELF3 | SERPINF1 |  | | ATP2C2 | | AP1S2 | |
| **CDH1** | SULT1B1 |  | KRT18 | SRGN |  | SCNN1A | LGALS1 |  | CEACAM6 | VCAM1 |  | | B3GNT3 | | ARMCX1 | |
| KLK5 | SNED1 |  | CDS1 | SPOCK1 |  | ST14 | PMP22 |  | TMPRSS4 | MAP1B |  | | BLNK | | ATP8B2 | |
| SPRR1B | PTX3 |  | THBD | PRR16 |  | ESRP1 | MSN |  | CLDN7 | TCF4 |  | | BSPRY | | ATP8B3 | |
| TP73L | **DCN** |  | NEFM | DLC1 |  | S100A14 | GLYR1 |  | TACSTD2 | SRPX |  | | C11orf52 | | AXL | |
| LAMA3 | COL6A2 |  | RPS6KA1 | BIN1 |  | CLDN7 | MAP1B |  | CLDN3 | EMP3 |  | | C19orf21 | | BDNF | |
| COL17A1 | GGTLA1 |  | SMPDL3B | RGL1 |  | ERBB3 | AP1S2 |  | EPCAM | DPT |  | | C1orf106 | | BICC1 | |
| FST | NR2F1 |  | ABCA12 | IGFBP4 |  | RBM47 | GJA1 |  | SPINT1 | CALD1 |  | | C1orf116 | | BNC2 | |
| ALDH1A3 | HHIP |  | RHOD | PVRL3 |  | SPINT1 | DENND5A |  | TSPAN1 | PTGIS |  | | C1orf210 | | BVES | |
| SFRP1 | GREM1 |  | KRT14 | CDH11 |  | ELF3 | C12orf24 |  | PLS1 | VIM |  | | C1orf34 | | C10orf38 | |
| KRT14 | GPM6B |  | PRKCH | OLFML3 |  | CLDN4 | TPM2 |  | TMEM30B | CD163 |  | | CCDC64B | | C10orf56 | |
| **IL1B** | STXBP6 |  | ZBED2 | MMP2 |  | PRSS8 | TUBB6 |  | PRR15L | C1R |  | | CD24 | | C16orf45 | |
| FXYD3 | NOV |  | C10ORF10 | MYL9 |  | SH3YL1 | SRPX |  | KRT8 | FBN1 |  | | CDH1 | | C1S | |
| CKMT1B | IGSF4B |  | LRRC1 | COL5A2 |  | EHF | ANK2 |  | ST14 | FN1 |  | | CDH3 | | C9orf19 | |
| **CD24** | NTSR1 |  | STAP2 | CTGF |  | LCN2 | CHN1 |  | RBM47 | FXYD6 |  | | CDS1 | | CAP2 | |
| ANXA8 | KCNMA1 |  | JUP | PLEKHC1 |  | JUP | SH2B3 |  | S100A14 | IGF1 |  | | CEACAM5 | | CCL2 | |
| **S100A8** | SDC2 |  | IL4R | ZEB1 |  | VAMP8 | LEPRE1 |  | C1orf106 | NAP1L3 |  | | CEACAM6 | | CDH11 | |
| KRT17 | PDGFRL |  | PERP | ROR1 |  | KRT8 | ETV1 |  | NQO1 | MRC1 |  | | CGN | | CDH2 | |
| SAA1 | **WNT5A** |  | FGFBP1 | PTGER2 |  | C1orf106 | SOBP |  | TOX3 | QKI |  | | CKMT1B | | CDH4 | |
| KLK7 | CLEC3B |  | MYO1D | CHN1 |  | KRT7 | AKAP12 |  | PTK6 | MS4A4A |  | | CLDN4 | | CHN1 | |
| DSP | **ABCA6** |  | FAT2 | PMP22 |  | DSP | TGFB1I1 |  | TFF1 | DCN |  | | CLDN7 | | CLDN11 | |
| KIBRA | COL8A1 |  | WWC1 | TRAM2 |  | CDS1 | SERPINE1 |  | CLDN4 | LOX |  | | CNKSR1 | | CLIP3 | |
| PRKCZ | EDNRA |  | FZD3 | TAGLN |  | ITGB4 | SOAT1 |  | GPRC5A | RECK |  | | CNTNAP2 | | CMTM3 | |
| TMEM30B | FADS2 |  | ZNF165 | TNFAIP6 |  | TMPRSS4 | COL5A2 |  | TJP3 | ANK2 |  | | CTAGE4 | | COL12A1 | |
| ANXA3 | LOXL1 |  | SNCA | CREB3L1 |  | LSR | LHFP |  | KRT18 | LY96 |  | | DAPP1 | | COL1A2 | |
| PI3 | EBF |  | KIAA1815 | UGDH |  | SORL1 | CEP170 |  | MAP7 | ZFPM2 |  | | DENND2D | | COL3A1 | |
| ZD52F10 | SERPINE1 |  | PRSS8 | HAS2 |  | GRHL2 | POPDC3 |  | CKMT1A | CSRP2 |  | | DMKN | | COL4A1 | |
| FBXO2 | FAM26B |  | SH2D3A | DNAJB4 |  | PPL | TRPC1 |  | ESRP1 | EFEMP1 |  | | DSC2 | | COL5A1 | |
| PHACTR3 | IGFBP4 |  | GNAL | CDKN2C |  | C1orf116 | KDELC1 |  | MUC1 | RARRES2 |  | | DSP | | COL5A2 | |
| S100P | SPOCK |  | BIK | CCDC92 |  | TSPAN1 | MYL9 |  | SPINT2 | PTPRC |  | | EHF | | COL6A1 | |
| CCND2 | PPP1R3C |  | CDH3 | WNT5A |  | MAP7 | BAG2 |  | ESRP2 | PLEKHO1 |  | | ELF3 | | CPA4 | |
| CNTNAP2 | PLEKHG4 |  | KIAA0888 | IGFBP3 |  | SLPI | FSTL1 |  | CDS1 | RGS2 |  | | ELF5 | | CTGF | |
| RAB25 | FBLN1 |  | KRT5 | PPM1D |  | TOX3 | MXRA7 |  | PPAP2C | F13A1 |  | | EPN3 | | CYBRD1 | |
| C1ORF116 | LOC51334 |  | GJB3 | FILIP1L |  | ARHGAP8 | GFPT2 |  | CEACAM7 | JAM2 |  | | EPPK1 | | DAB2 | |
| EPN3 | CHST2 |  | KIAA0040 | PDGFC |  | F11R | RECK |  | TTC39A | CHRDL1 |  | | ERBB3 | | DFNA5 | |
| AJAP1 | COPZ2 |  | CELSR2 | TBX3 |  | LAD1 | TMEFF1 |  | OVOL2 | TUBA1A |  | | ERP27 | | DIO2 | |
| ZBED2 | MAP1B |  | F11R | XYLT1 |  | GPX2 | PTRF |  | EHF | AP1S2 |  | | FA2H | | DKK3 | |
| TPD52L1 | AOX1 |  | NUP62CL | FAP |  | CTSH |  |  | AP1M2 | MYLK |  | | FAAH2 | | DLC1 | |
| F11R | RGL1 |  | SERPINB1 | DPT |  | GPR56 |  |  | CEACAM5 | DDR2 |  | | FAM110C | | DOCK10 | |

50 E and M-specific genes signatures were derived from the indicated publications. E/M signatures are a composite from the respective most extreme 25 E and 25 M genes. Bold genes amongst HMLER-derived E and M signatures were amongst the genes used for single cell qPCR analysis.
